# From vitality to vulnerability: the impact of oxygen on cardiac function and regeneration

**DOI:** 10.20517/jca.2024.05

**Published:** 2024-02-21

**Authors:** Dogacan Yucel, William T. Pu

**Affiliations:** Department of Cardiology, Boston Children’s Hospital, Boston, MA 02115, USA.

The heart pumps over 7,000 liters of blood daily and, in the process, consumes over 6 kg ATP. To fulfill this prodigious demand, the heart relies on mitochondrial oxidative phosphorylation, in which oxygen and appropriate substrates are used to generate ATP. While oxygen and oxidative phosphorylation are essential for the heart to meet its metabolic demands, at the same time, they limit cardiomyocyte proliferation and the heart’s regenerative capacity. Reactive oxygen species (ROS) can be a by-product of oxidative phosphorylation. Excessive ROS is toxic for cells, at least in part, by damaging DNA and activating the DNA damage response^[1]^. A series of studies by Hesham Sadek’s team at the University of Texas Southwestern have highlighted the double-edged sword that oxygen poses to cardiomyocytes by tying the postnatal elevation of cardiomyocyte oxygen consumption and oxidative phosphorylation to cardiomyocyte cell cycle exit^[2]^ [Figure 1]. In a recent publication in *The Journal of Cardiovascular Aging*^[3]^, the Sadek group extended this body of work by identifying HIF2α as a key oxygen-sensitive transcriptional regulator of cardiomyocyte cell cycle activity^[3]^.

In the relatively hypoxic intrauterine environment, cardiomyocytes mainly utilize glycolysis as the primary source of energy^[4]^. At birth, the higher extra-uterine oxygen tension combined with other environmental and hormonal changes drive a metabolic shift in cardiomyocytes towards oxidative phosphorylation and greater oxygen consumption. This shift is accompanied by the loss of cardiomyocyte proliferative capacity^[5]^. The Sadek group’s prior work demonstrated a causal link between this switch to oxidative phosphorylation and loss of cardiomyocyte proliferative capacity, as ROS generated by cardiomyocyte oxidative phosphorylation damages DNA, resulting in activation of DNA damage response pathways and inhibition of cell cycle activity^[2]^. Conversely, depriving cardiomyocytes of oxygen allows them to re-enter the cell cycle^[6]^.

Ali e*t al.*’s recent study published in *The Journal of Cardiovascular Aging* centers on the hypothesis that hypoxia-induced gene upregulation plays a direct role in regulating cardiomyocyte proliferation^[3]^. The transcription factors hypoxia-inducible factor 1-alpha and 2-alpha (HIF1α and HIF2α) are well-known mediators of oxygen-regulated transcription. In normoxic conditions, these proteins undergo oxygen-dependent hydroxylation and subsequent proteasomal degradation. Hypoxia stabilizes the proteins and allows them to activate transcription of their target genes. Ali *et al.* demonstrated that adult cardiomyocyte cell cycle activity induced by hypoxia required *Hif2α* but not *Hif1α*. The impact of *Hif2α* inactivation on cardiac function was not disclosed. Cardiomyocyte-specific overexpression of a modified version of HIF2α that is resistant to oxygen-induced degradation (Hif2α^OE^) increased cardiomyocyte proliferation and the number of cardiomyocytes recovered from dissociated hearts. The number of cardiomyocytes labeled by a single color in the mosaic analysis with double markers (MADM) system^[7]^, in which single-color cells are thought to be produced only by cell division, increased by 2.5-fold, providing additional evidence for induction of cell cycle activity by Hif2α^OE^. Cell size, overall heart size, and heart function were not significantly affected. Hif2α^OE^ decreased oxidative DNA damage and suppressed the DNA damage response pathway, which the group had previously shown to inhibit cardiomyocyte cell cycle activity. Additionally, when initiated one week after experimental myocardial infarction, Hif2α^OE^ demonstrated protective effects on myocardial function compared to controls, suggesting potential therapeutic applications for activation of the HIF2α pathway. However, it was not determined if this beneficial effect was mediated by increased cardiomyocyte proliferation or alternative effects of Hif2α^OE^.

A growing body of literature from Sadek’s group^[2,3,6,8]^ and others^[9,10]^ has highlighted the intricate link between oxygen levels, metabolic shifts, and cardiomyocyte cell cycle regulation. These findings are promising and suggest that inducing hypoxia or targeting hypoxia- and metabolism-related pathways are potential strategies to promote therapeutic cardiac regeneration. As is often the case for new findings, several points need to be further clarified. Potential pathological remodeling resulting from candidate interventions should be considered, as modifications of some metabolic pathways in cardiomyocytes in mice have led to hypertrophy and pathological changes in addition to cardiomyocyte proliferation^[10]^. Thus, the safety of Hif2α^OE^ as a therapeutic strategy for heart failure requires further studies. In addition, current techniques have limitations in providing quantitative, robust, and reproducible measurements of cardiomyocyte proliferation and the extent of cardiac regeneration. The number of histone H3-stained nuclei in cells that are co-stained for cardiac troponin T, while frequently used as in the present study, may not accurately identify cardiac myocytes^[11]^. Additional proliferation markers would increase the accuracy and reliability of cardiomyocyte proliferation measurements. It is also generally recognized that pH3 or EdU label cells in the M and S phases of the cell cycle and may not provide a full picture of cardiomyocyte proliferation due to cardiomyocyte endoreplication. To their credit, the authors have used the MADM genetic system, which is a state-of-the-art method for assessing cardiomyocyte proliferation in adult mice. Although MADM detected relatively greater myocyte proliferation in Hif2α^OE^ cardiomyocytes, the observed 1% single-color cardiomyocytes over two weeks under baseline conditions is higher than the currently accepted cardiomyocyte proliferation rate of humans and mice^[12,13]^. Lastly, like any new findings, it will be important that key results are replicated in additional independent studies.

In conclusion, the hypothesis linking hypoxia to cardiomyocyte proliferation is intellectually satisfying and offers promising avenues for cardiac regeneration. However, significant challenges and discrepancies exist, which must be surmounted so that these exciting initial findings can pave the way for safe and effective therapies.

## Figures and Tables

**Figure 1.**
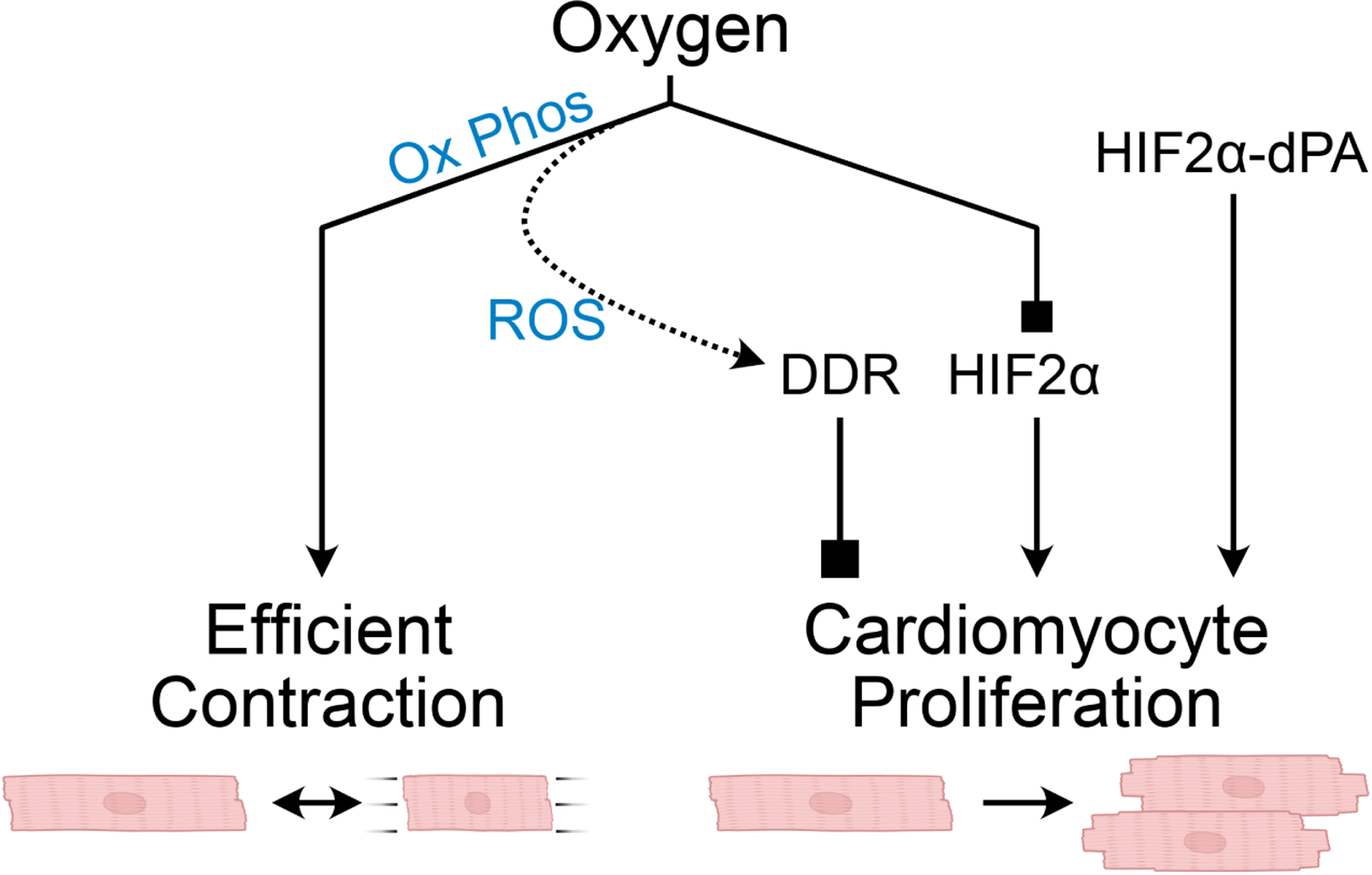
The double-edged sword of oxygen in cardiomyocyte function and proliferation. Oxygen is required for efficient energy production and contraction of cardiomyocytes, but it blocks cardiomyocyte proliferation by inducing the DNA damage response (DDR) and by inactivating HIF2α. Overexpression of HIF2α-dPA, an oxygen resistant form of HIF2α, promotes cardiomyocyte proliferation and improved myocardial outcome after experimental MI. Graphics from BioRender.com.
